# Online Information as a Double-Edged Sword: A Multilevel Analysis of Vaccination Skepticism Based on ISSP 2021

**DOI:** 10.1007/s10900-026-01599-2

**Published:** 2026-07-16

**Authors:** Alexander Helbing, Juliane Heise, Sophie Redlin, Peter Kriwy

**Affiliations:** Department of Sociology, University of Technology Chemnitz, Thüringer Weg 9, 09126 Chemnitz, Germany

**Keywords:** Vaccine hesitation, Multi-level analysis, Digital health, Vaccines, Public health

## Abstract

This study examines the relationship between internet use and vaccine scepticism, focusing on the ambivalent role of digital information environments and digital health literacy (DHL). While the internet provides easy access to vaccination-related information, frequent internet use may also increase exposure to misinformation and disinformation. Accordingly, both positive and negative effects of internet-based information seeking are hypothesized. In contrast, higher levels of DHL are expected to reduce vaccine scepticism. The study also investigates the influence of healthcare system quality, measured through both subjective satisfaction with the healthcare system and an objective indicator of healthcare quality. The hypotheses are tested using data from the International Social Survey Programme (ISSP) 2021, employing multilevel logistic regression models based on *N* = 48,538 respondents. The results reveal a non-linear relationship between internet use and vaccine scepticism. Individuals without internet access as well as those who search very frequently for vaccination-related information online exhibit higher levels of scepticism. In addition, low DHL is consistently associated with greater vaccine scepticism, suggesting that the ability to critically evaluate online health information is as important as access itself. Furthermore, dissatisfaction with the healthcare system and lower national healthcare quality are linked to more sceptical attitudes toward vaccination. These findings highlight the internet as both an opportunity for health education and a potential source of misinformation. Public health strategies should therefore ensure equitable access to reliable vaccination information while strengthening digital health literacy and individuals’ critical evaluation skills.

**Supplementary Information:**

The online version contains supplementary material available at https://doi.org/10.1007/s10900-026-01599-2.

## Introduction

Vaccine hesitancy remains a major challenge for public health, as it can undermine vaccination coverage, reduce the effectiveness of immunization programs, and it hinders the building of herd immunity [1–4]. While vaccines are among the most effective tools for preventing infectious diseases and are estimated to have prevented 154 million death since 1974 [5], public confidence in their safety, efficacy, and necessity varies considerably across populations and contexts [6]. Vaccine hesitancy itself can be defined as refusal or reluctance to get vaccinated despite the availability of vaccines [1, 7]. However, before such hesitation is reflected in vaccination behaviour, it may be shaped by vaccine scepticism as an attitudinal construct.

There is already a substantial body of research identifying multiple determinants of vaccination hesitancy and vaccination uptake in general [8–11]. Sociodemographic variables such as gender, age, and education have been shown to have a significant influence on vaccination uptake [12–14]. In addition, social and cultural factors, such as social networks and social pressure, can also influence the decision to vaccinate [8, 15–18]. Vaccination decisions are therefore not solely based on individual medical considerations but are embedded in broader social, cultural, and informational contexts. However, one of the most cited reasons for vaccination hesitancy are perceptions regarding vaccine safety, effectiveness, and necessity, which can be summarized as scepticism towards vaccines [8, 10, 11, 19–21]. Understanding the attitudinal foundations of vaccine scepticism is therefore essential for explaining why individuals may develop doubts about vaccination and, ultimately, become reluctant to vaccinate.

The aim of this study is to explain the existence and formation of vaccine scepticism. Although vaccine scepticism has received less research attention than vaccine hesitancy and vaccination behaviour, scepticism toward vaccines has nevertheless been examined in previous studies. Some studies have focused on specific population groups, such as healthcare professionals, parents, or the general public [22–24]. Other studies have investigated attitudes toward vaccination-related public health measures, such as mandatory vaccination policies, rather than attitudes toward vaccination itself [25, 26]. Reported determinants of scepticism include factors such as educational background, moral values, and conspiratorial thinking [22, 27, 28]. However, the present study focuses specifically on the role of the internet in shaping scepticism toward vaccination.

The internet is an important source for health information and plays a significant role in shaping attitudes regarding benefits and risks of health-related behaviour [29–32]. Prevention awareness campaigns, advertising, and social media are among several ways in which the internet can shape public perceptions of public health measures like vaccination [33–35]. With rapid digitalisation, it is increasingly important to examine these interrelationships. In particular, both the intensity of internet use (time spent online) and the types of use (consumption via YouTube, Instagram or scientific publications) have been identified as potentially relevant to vaccination decisions [16, 19, 36, 37].

The role of the internet as an information source, however, is ambivalent. On one hand, it provides easy access to important information, such as tips for a healthy lifestyle, the interpretation of early illness symptoms, and guidance on how to live with illness or chronic diseases [37, 38]. It helps people to better understand vaccination, gain more knowledge and to fight vaccine hesitancy [29, 31, 33]. On the other hand, the digital environment also facilitates the spread of misinformation, as any type of content, whether accurate or false, can reach large audiences quickly and is often amplified by algorithmic mechanisms [21, 39–43]. Online environments may also facilitate exposure to conspiracy theories that are associated with negative vaccine attitudes [15, 42, 44]. Research shows that internet accessibility is associated with large increases in vaccination hesitancy [21, 39]. In addition, people who use or trust the internet and social media as a source of information are more hesitant when it comes to vaccination [16, 19, 36, 40].

Taken together, both the absence of internet access and excessive internet use for information seeking may be associated with higher levels of vaccine sceptics. Having no internet access may lead to a lack of education about vaccinations, when to get them, and why they are necessary. Conversely, excessive internet use increases the risk of encountering misinformation and disinformation. This suggests a u-shaped relationship, whereby both very low and very high levels of internet use are associated with higher levels of vaccination scepticism. This leads to the following hypotheses.

H1: Both very low and very high levels of internet-based information seeking are associated with higher levels of vaccination scepticism.

The frequency of internet information seeking may not be sufficient to explain individual differences in vaccination attitudes. Instead, digital health literacy (DHL) may also play an important role. DHL refers to an individual’s ability to find, assess, process, and critically evaluate health information available online [45]. Previous research has identified associations between DHL and vaccination behaviour [46, 47]. Individuals with high levels of digital health literacy are, therefore, expected to be more resistant to misinformation and false claims, even if they frequently use the internet to search for health-related information. Based on these considerations, the following hypothesis is proposed:

H2: Individuals with higher digital health literacy are less likely to report higher levels of vaccination scepticism.

Trust in vaccination and individuals’ willingness to receive vaccines are likely to be shaped not only by individual characteristics but also by the quality of the healthcare system [48]. A high-quality healthcare system should actively promote and support vaccination education while ensuring that vaccinations are readily accessible to the majority of the population. Easy access to vaccination services enables individuals to gain personal experience with vaccinations, which may reduce vaccine-related fears and foster more positive attitudes toward vaccination. Consequently, improved accessibility and education may contribute to greater public confidence in vaccines and higher vaccination coverage.

Studies show that the quality of a healthcare system depends not only on objective features but also on individuals’ subjective perceptions [49]. A healthcare system that is perceived as high quality may increase confidence in medical recommendations and public health measures, including vaccination. Conversely, low subjective quality in the healthcare system may foster doubts about the safety, effectiveness, or necessity of vaccines and may therefore contribute to higher levels of vaccination scepticism. Subjective quality in the healthcare system does not necessarily correspond to the objective quality of healthcare provision. Individuals may assess healthcare institutions as being of low quality even in countries with comparatively high healthcare access and quality, while individuals in lower-quality systems may still report high levels of trust. Therefore, this study distinguishes between a subjective individual-level measure of quality regarding the healthcare system and objective country-level indicators of the broader structural context. Consequently, the following hypotheses are proposed:

H3a: Higher *subjective* healthcare system quality is associated with lower levels of vaccination scepticism.

H3b: Higher *objective* healthcare system quality is associated with lower levels of vaccination scepticism.

This study contributes to research on vaccine hesitancy by shifting the focus from vaccination behaviour to vaccine scepticism as an attitudinal precursor of hesitancy. While previous studies have primarily examined vaccination uptake, delay, or refusal, less is known about why individuals develop sceptical attitudes toward the safety, effectiveness, or necessity of vaccines in the first place. Addressing this question is important because sceptical attitudes may emerge before they translate into behavioural reluctance or refusal to vaccinate. By examining individual-level factors, such as internet-based information seeking, digital health literacy, and trust in the healthcare system, alongside country-level indicators, such as healthcare system quality and income inequality, this study aims to provide a more comprehensive understanding of the conditions under which vaccination scepticism develops.

## Methods

### Study Sample

To test these hypotheses, this study uses data from the International Social Survey Programme (ISSP) 2021 dataset. The ISSP 2021 focuses on health and healthcare, providing relevant items for this study regarding vaccination attitudes. A total of 32 countries participated, with 30 countries included in the original dataset. Separate datasets exist for the United Kingdom (UK), Greece, Spain [50], and Sweden [51]. The data from Spain and Sweden are combined with the original dataset, while the data from the UK and Greece are not included due to major differences in data collection and data quality. Mexico has the smallest sample size (*n* = 1,001), while Switzerland has the largest (*n* = 3,349). For a complete overview of the included countries and sample sizes, see supplementary material (S1). In total, *N* = 48,538 respondents participated in the survey between 2021 and 2024. The mean age of respondents is 49.76 years (standard error = 17.49). 53.70% of respondents identify as female and 46.30% as male. A descriptive sample analysis is presented in Tables S2.1 and S2.2 in the supplementary material.

### Operationalization

Two dependent variables are used to capture vaccine scepticism: subjective harmfulness of vaccination and preference for natural immunity. Respondents are asked whether they agree or disagree with the following statements: ‘Overall, vaccinations do more harm than good’ and ‘It is better to develop immunity by getting ill than by having a vaccination.’ Responses measured on a five-point Likert scale but are operationalised as binary variables (0 = neither agree nor disagree, disagree and strongly disagree, 1 = agree and strongly agree). Individuals who answered “I don’t know” are also assigned to the reference category, so that only active agreement with the statements is coded as 1. Using both variables increase the validity of the study and enables testing for consistency in effects.

The independent variables are internet access, frequency of searching for vaccination-related information and DHL. For respondents with no internet access the missing-indicator approach is applied [52], constructing a binary variable indicating internet access (0 = has internet access, 1 = has no internet access,). This variable is based on a question that assessed how frequently respondents use the internet for health-related reasons in general. Respondents are given a 6-point scale, and the option “I do not have access to the internet”. For the frequency of internet use regarding vaccination-related information and DHL variables, missing values resulting from a lack of internet access are imputed using the mode of the distribution. Frequency of internet use regarding vaccination-related information is measured as a categorical variable (0 = Never, 1 = seldom, 2 = sometimes, 3 = often, 4 = very often). For DHL respondents are asked whether they agree to the following statement: “It is not easy to distinguish between reliable and unreliable health information on the internet.” Agreement is coded as low DHL, while disagreement is coded as high DHL. A neutral response category is included as the reference category (0 = neither high nor low DHL, 1 = low DHL, 2 = high DHL).

There are some country-specific differences in the measurement and coding of internet-related variables. For the original variable on which the internet access dummy is based, respondents in Hungary are not filtered out for the subsequent question on the frequency of searching for vaccination-related information. In Poland, the corresponding question uses a 7-point response scale instead of the 6-point. Moreover, although respondents who answer “never” to the original internet use variable are filtered out in the Polish dataset, they are retained for the present analysis and recoded as “never” for the vaccination information-seeking variable. For DHL measure, Poland also includes the response option “I don’t know, I don’t use the internet,” which results in a higher number of missing values.

This study includes two variables measuring the quality of the healthcare system: a subjective individual-level measure and an objective macro-level measure. The subjective individual-level variable captures respondents’ satisfaction with their country’s healthcare system (0 = neither high nor low satisfaction, 1 = low satisfaction, 2 = high satisfaction). The objective macro-level variable is based on the Healthcare Access and Quality Index (HAQ) [53], which measures the accessibility and quality of healthcare at the country level. The HAQ is originally measured as a continuous variable ranging from 0 (lowest quality) to 1 (highest quality) (M = 0.73, SD = 0.20). Due to violations of the linearity assumption, the variable is categorized into terciles (0 = medium HAQ, 1 = low HAQ, and 2 = high HAQ).

Finally, several control variables are included. To account for sociodemographic factors, gender (0 = male, 1 = female), age (metric), and education are used. Education is measured using the International Standard Classification of Education (ISCED 2011) which is compromised into three categories (0 = no formal education and primary education, 1 = upper secondary and post-secondary non-tertiary education, 2 = short-cycle tertiary, lower-level tertiary, upper-level tertiary, PhD, and post tertiary specialization). Furthermore, subjective health status is considered (0 = excellent and very good health, 1 = good health, 2 = fair and poor health). Additionally, the Gini coefficient (Gini) is included as a second country-level variable to capture structural differences between countries [21, 41, 54, 55]. The Gini is originally a metric variable (M = 0.51, SD = 0.11) measuring income inequality within a country, ranging from 0 (perfect equality, where income is equally distributed) to 1 (maximum inequality, where a single individual holds all income while all others have none). Income inequality may be relevant for vaccination scepticism because more unequal societies can be characterized by lower social cohesion, unequal access to resources, and lower trust in institutions. Thus, vaccination scepticism may also be shaped by broader contextual conditions related to social inequality. Due to concerns regarding linearity, it is also categorized into terciles (0 = medium Gini, 1 = low Gini, 2 = high Gini). Data collection took place from 2021 to 2024. Due to the COVID-19 pandemic and the resulting vaccination campaigns, contextual influences may be present. Therefore, a variable controlling for survey year is added. The full operationalisation is presented in Table S3 in the supplementary material.

### Statistical Analysis

Two models are estimated for each dependent variable (Model 1: subjective harmfulness of vaccination; Model 2: preference for natural immunity). Given the hierarchical structure of the data, with individuals nested within countries, multilevel logistic regression is employed. The intra-class correlation coefficients (ICC) amount to ICC_Model1_ = 0.14 and ICC_Model2_ = 0.18 indicating a relatively high proportion of between-country variance and thereby supporting the use of a multilevel approach. To check for potential multicollinearity in the logistic regression model, we assess the variance inflation factor (VIF) coefficients. The overall highest VIF coefficient observed is 1.54 and therefore unproblematic . All analyses are conducted in R (version 4.6.0) using the lme4 package (version 1.1–38) for multilevel logistic regression. The results are shown in odds ratios (OR) with corresponding standard errors (SE). Statistical significance is assessed at α = 0.05, and cases with missing values are excluded using listwise deletion.

## Results

**Table 1:** Multilevel logistic Regression on vaccination attitudes

	Model 1		Model 2	
	‘Overall, vaccinations do more harm than good’ (0 = disagree, 1 = agree)		‘It is better to develop immunity by getting ill than by having a vaccination’ (0 = disagree, 1 = agree)	
Predictors	OR	SE	OR	SE
*Internet access*				
Yes	*Ref.*	*Ref.*	*Ref.*	*Ref.*
No	1.14 *	0.07	1.12 *	0.06
*Frequency internet use*				
Never	*Ref.*	*Ref.*	*Ref.*	*Ref.*
Seldom	0.96	0.05	0.87 ***	0.04
Sometimes	0.84 ***	0.03	0.79 ***	0.03
Often	0.82 ***	0.03	0.81 ***	0.03
Very often	1.23 **	0.09	1.08	0.07
*Digital health literacy*				
Mid	*Ref.*	*Ref.*	*Ref.*	*Ref.*
Low	1.13 ***	0.04	1.18 ***	0.04
High	1.08	0.05	1.05	0.05
*Satisfaction healthcare system*				
Neutral	*Ref.*	*Ref.*	*Ref.*	*Ref.*
Satisfied	0.79 ***	0.03	0.83 ***	0.03
Not satisfied	1.33 ***	0.06	1.32 ***	0.05
*HAQ*				
Mid	*Ref.*	*Ref.*	*Ref.*	*Ref.*
Low	1.19	0.15	1.19	0.14
High	0.72 *	0.11	0.82	0.11
*Gender*				
Male	*Ref.*	*Ref.*	*Ref.*	*Ref.*
Female	0.98	0.03	0.87 ***	0.02
Age (metric)	0.99 ***	0.00	0.99 ***	0.00
*Education*				
Primary education or less	*Ref.*	*Ref.*	*Ref.*	*Ref.*
Secondary education	0.78 ***	0.04	0.94	0.04
Tertiary education	0.59 ***	0.03	0.67 ***	0.03
*Subjective Health*				
Bad	*Ref.*	*Ref.*	*Ref.*	*Ref.*
Fair	0.81 **	0.06	0.83 **	0.06
Good	0.86 *	0.06	0.87 *	0.06
Very good	0.93	0.07	0.96	0.06
Excellent	0.96	0.08	1.08	0.08
*Gini*				
Mid	*Ref.*	*Ref.*	*Ref.*	*Ref.*
Low	0.95	0.15	1.18	0.19
High	1.23	0.17	1.08	0.18
*Survey year*				
2021	*Ref.*	*Ref.*	*Ref.*	*Ref.*
2022	1.26 *	0.12	1.29 *	0.13
2023	1.42 *	0.22	1.73 ***	0.25
2024	0.68	0.17	0.90	0.21
ICC	0.14		0.18	
N_Cluster_	32		32	
Observations	45,208		45,201	
Marginal R^2^/Conditional R^2^	0.08/0.16		0.05/0.19	

OR = odds ratio; SE = standard error; Ref.= reference category; empirical significance level (z-test, two-sided): ***p ≤ 0.001, **p ≤ 0.01, *p ≤ 0.05

Overall, 14.87% (*n* = 7,173) of respondents agreed that vaccinations do more harm than good. Similarly, 19.93% (*n* = 9,614) agreed that natural immunisation is better than vaccination. Spearman’s rank correlation indicated a strong positive association between the two variables (ρ = 0.61, *p* < 0.001). To calculate Spearman’s rank correlation, the ordinal versions of the variables are used, with “I don’t know” responses recoded into the “neither agree nor disagree” category. This substantial correlation provides evidence for convergent validity.

The results of the multilevel logistic regression analyses are presented in Table 1. The vaccine scepticism model yielded a conditional R² of 0.16, while the natural immunity model yielded a conditional R² of 0.19. Overall, these values indicate a moderate level of explanatory power for the multilevel models.

The variables related to internet use show mixed results. Individuals without internet access are significantly more likely to consider vaccines harmful and to prefer natural immunity over vaccination, although the observed effects are relatively small (Model 1: OR = 1.14, *p* < 0.05; Model 2: OR = 1.12, *p* < 0.05). Regarding the frequency of using the internet to obtain information about vaccinations, respondents who used the internet seldom, sometimes, or often have lower odds for vaccination scepticism than those who never used the internet for this purpose (Model 1: OR_Seldom_ = 0.96, *p* > 0.05; OR_Sometimes_ = 0.84, *p* < 0.001; OR_Often_ = 0.82, *p* < 0.001; Model 2: OR_Seldom_ = 0.87, *p* < 0.001; OR_Sometimes_ = 0.79, *p* < 0.001; OR_Often_ = 0.81, *p* < 0.001). Thus, more frequent internet use is generally associated with lower odds of vaccination scepticism, although the effect for the “seldom” category was not statistically significant in Model 1. In contrast, respondents who report using the internet very often showed higher odds of vaccination scepticism. However, this effect is statistically significant only in Model 1 (OR = 1.23, *p* < 0.01), whereas the corresponding effect in Model 2 is not significant (OR = 1.08, *p* > 0.05). This suggests that both not having internet access and not using the internet for information purposes, as well as very frequent internet use, are associated with more vaccination scepticism. In Addition, lower DHL is also leading to significantly more vaccine scepticism (Model 1: OR = 1.13, *p* < 0.001) and preference for natural immunity (OR = 1.18, *p* < 0.001). In contrast, high digital health literacy was not significantly associated with either outcome compared with the neutral reference category (Model 1: OR = 1.08, *p* > 0.05; Model 2: OR = 1.05, *p* > 0.05).

Regarding perceptions of the quality of the healthcare system, high subjective satisfaction is negatively associated with vaccination scepticism (Model 1: OR = 0.79, *p* < 0.001; Model 2: OR = 0.83, *p* < 0.001), whereas low subjective satisfaction shows a positive association (Model 1: OR = 1.33, *p* < 0.001; Model 2: OR = 1.31, *p* < 0.001). The objective quality of the healthcare system shows similar patterns: low quality is positively associated with vaccination scepticism (Model 1: OR = 1.19, *p* > 0.05; Model 2: OR = 1.19, *p* > 0.05), while higher quality is negatively associated with vaccination scepticism (Model 1: OR = 0.72, *p* < 0.05; Model 2: OR = 0.81, *p* > 0.05). However, only the effect for high quality in Model 1 is statistically significant.

The control variables show heterogeneous results. Women tend to hold slightly less vaccination scepticism (Model 1: OR = 0.98, *p* > 0.05; Model 2: OR = 0.87, *p* < 0.001). Age (Model 1: OR = 0.99, *p* < 0.001; Model 2: OR = 0.99, *p* < 0.001) and higher education levels (Model 1: ORS_econdary_= 0.78, *p* < 0.001; OR_Tertiary_ = 0.59, *p* < 0.001; Model 2: OR_Secondary_ = 0.94, *p* > 0.05; OR_Tertiary_ = 0.67, *p* < 0.001) are generally negatively associated with vaccination scepticism. At the same time, better self-reported health status is also negatively associated with vaccination scepticism (Model 1: OR_Fair_ = 0.81, *p* < 0.01; OR_Good_ = 0.86, *p* < 0.05; OR_Very good_ = 0.93, *p* > 0.05; OR_Excellent_ = 0.96, *p* > 0.05; Model 2: OR_Fair_ = 0.84, *p* < 0.01; OR_Good_ = 0.87, *p* < 0.05; OR_Very good_ = 0.96, *p* > 0.05; OR_Excellent_ = 1.08, *p* > 0.05). However, most effects are not statistically significant, and excellent health status even shows a positive association in Model 2. In contrast, the GINI coefficient does not show a significant or notable results.

Both models also include the survey year as a control variable. This shows that vaccination scepticism changes throughout time (2021–2024). Compared to 2021, vaccine scepticism increased in 2022 (Model 1: OR = 1.26, *p* < 0.05; Model 2: OR = 1.29, *p* < 0.05) and especially in 2023 (Model 1: OR = 1.42, *p* < 0.05; Model 2: OR = 1.73, *p* < 0.001). The effects for 2024 are not significant in both models and show negative associations with vaccine scepticism (Model 1: OR = 0.68, *p* > 0.05; Model 2: OR = 0.90, *p* > 0.05). 

## Discussion

The aim of this study is to examine the role of internet use in explaining vaccine scepticism. We argue that internet as a source for information can have both positive and negative effects. When individuals do not have internet access or do not use the internet to obtain information, they may miss important information regarding vaccinations. This may limit access to relevant information and may be associated with greater vaccine scepticism. However, frequent internet use can also increase exposure to misinformation and disinformation about vaccinations. By using a large cross-national dataset and applying multilevel logistic regression, the analysis provides evidence for these ambivalent associations. Both having no internet access at all and using the internet very frequently are associated with higher vaccine scepticism. In addition, low DHL is associated with higher vaccine scepticism, strengthening the argument that not only access itself, but also how people process and evaluate information, plays an important role. Therefore, the findings provide partial support for H1. Hypothesis H2 cannot be directly supported, as we do not find the postulated negative effect of high DHL. Nevertheless, we find a significant positive effect for low DHL, indicating that particularly low levels of DHL predict vaccine scepticism.

The findings are consistent with previous research. McRee et al. showed that the internet can contribute to higher levels of knowledge about vaccination [31]. On the other hand, frequent internet use may also increase exposure to misinformation and disinformation [12, 33, 41, 56]. While most studies focus on vaccination behaviour and vaccine hesitancy, a few studies also find a negative effect of internet use on vaccination scepticism [21, 40]. Online platforms may repeatedly expose users to content that corresponds with their previous searches and existing attitudes, thereby reinforcing pre-existing beliefs. This ambivalent effect is important because it should be taken into account when designing future public health measures. It highlights that the effectiveness of health education depends on how individuals use the internet and therefore should be tailored to specific target groups. On the one hand, it is important to ensure that individuals without internet access receive sufficient information about vaccinations for example through healthcare professionals, pharmacies or public health services. On the other hand, frequent internet users should be educated about the risks of misinformation and disinformation related to vaccination. Such measures should not only provide accurate information but also strengthen individual´s ability to identify reliable sources and critically evaluate online content. Therefore, the internet can be seen as both an opportunity and a challenge [29, 33].

The findings further emphasize the importance of the quality of the healthcare system. Healthcare system quality is assessed using both subjective satisfaction with the healthcare system and the objective HAQ indicator. The results show that low satisfaction with the healthcare system is associated with greater vaccine scepticism. Therefore, H3a is supported. This finding is consistent with previous research, which has identified satisfaction with, and particularly trust in, the healthcare system as an important determinant of vaccination attitudes and uptake [12, 17, 19]. Individuals who are dissatisfied with the healthcare system may also perceive information and recommendations provided by healthcare institutions as less credible. However, satisfaction and trust are not identical concepts. Higher HAQ is associated with slightly lower vaccine scepticism. Therefore, H3b is also supported. This effect may be explained by the fact that countries with higher quality healthcare systems are more able to educate people about vaccination. Another possible explanation is that these countries have better vaccination programmes. A well-developed vaccination programme may contribute to higher vaccination rates and therefore greater individual experience with vaccinations. As a result, vaccinations are less likely to be perceived as an unknown risk, making the associated risks easier to assess. This was shown during COVID-19, where the novelty of mRNA vaccines was associated with increased vaccine hesitancy due to their unfamiliarity [57, 58].

The results for the control variables are discussed in the following section. Overall, studies find a negative association between age and vaccine hesitancy, meaning that older individuals are generally less hesitant and more likely to be vaccinated [11, 13, 14]. We can reproduce this effect regarding vaccination scepticism. One possible explanation could be that older people are more susceptible to infectious diseases and therefore perceive a higher personal risk. Regarding gender, research is often reporting a negative effect of being female on vaccination uptake or attitudes [6, 12, 16, 20]. Other studies find a negative effect of being male [10, 13]. In our study, females tend to show lower levels of scepticism toward vaccinations. This effect is, however, only observed for the statement that natural immunization is better than vaccination. One possible explanation for this finding may be masculinity norms, suggesting that men are more likely to believe their bodies are strong enough to resist illness and therefore perceive less need for medical prevention [59, 60]. Our results also support earlier studies and show that education seems to protect against vaccine hesitancy [13, 20, 22, 61]. The reasoning behind this is that higher education can lead to higher DHL [62].

The study also provides information on time-related differences. The findings show that vaccine hesitancy increased after 2021 but decreased again in 2024 in both models, although the effect is not statistically significant. During the COVID-19 pandemic, vaccination was widely discussed in public discourse. In addition, the rapid development of COVID-19 vaccines and the deployment of novel vaccine technologies, particularly mRNA-based vaccinations , may have influenced public attitudes and vaccine hesitancy [57, 58, 63]. This is a crucial point, as it suggests that scepticism toward a specific vaccine type (e.g., mRNA vaccines) may generalize, leading to broader vaccine hesitancy. This is particularly relevant because the ISSP question does not specifically refer to mRNA or COVID-19 vaccines, but to vaccination in general. This phenomenon reflects therefore potential spillover effects. However, the effect appears to decease by 2024, suggesting that it is specific to the COVID-19 pandemic and not long-term in nature. However, future research is necessary to examine whether vaccination hesitancy has lastingly increased due to COVID-19.

We do not find any notable effect of the Gini coefficient. This is in line with previous studies, which find macro-level country effects for other variables but no significant association between income inequality, as measured by the Gini coefficient, and the outcome of interest [17].

Some limitations of the study are to be discussed. First, the cross-sectional study design does not allow for causal interpretation. For example, we cannot determine whether individuals developed vaccination scepticism because they encountered misinformation online, or whether they already held negative attitudes and actively sought out information that confirmed their existing beliefs. Another limitation is the measurement of DHL using a single-item indicator based on self-reporting. A single item may not fully capture the multidimensional nature of digital health literacy, which may lead to reduced validity and potential measurement error. Also, self-reported DHL may be affected by response bias. Respondents may overestimate their ability to evaluate whether information is correct. This could result in individuals with relatively low DHL classifying themselves as having high DHL. Nevertheless, our results indicate meaningful effects for low DHL (leading to higher vaccine scepticism). Therefore, our estimates are likely conservative, and the true effects may be stronger. Finally, countries differ in survey modes, which may introduce mode effects and potentially influence how respondents interpret and answer the questions.

## Conclusion

The ISSP 2021 data and the multilevel analysis indicates a significant association between online information seeking and scepticism towards vaccines. The findings presented a non-linear relationship between internet use and vaccine attitudes. Moderate internet use leads to lower vaccine scepticism, whereas very frequent internet use leads to higher scepticism. Therefore, internet use can have both positive and negative effects on vaccine scepticism. The positive or negative impacts depend on intensity and type of online use. Future research should focus on this effect and examine potential interaction effects, particularly those that may reduce susceptibility to misinformation and disinformation when the internet is used frequently as an information source. Further research is needed not only on the quantity but also on the quality of information. Information-seeking behaviour differs depending on the type of source, such as social media, health-related websites, or official websites of scientific institutions.

Additionally, both subjective and objective evaluations of the healthcare system are important factors. However, the associations are stronger and more consistent for the individual-level satisfaction measure. Future research should further investigate this difference and examine whether subjective or objective assessments play a more important role in shaping vaccination attitudes. This is highly relevant for future public health strategies, as it suggests that improving the healthcare system alone may not be sufficient. Rather, it is also necessary to strengthen public awareness and information campaigns in order to foster positive perceptions and attitudes.

## Supplementary Information

Below is the link to the electronic supplementary material.

Supplementary Material 1

## Data Availability

The data that support the findings of this study are available from GESIS Leibniz Institute for the Social Sciences (https://doi.org/10.4232/5.ZA8000.2.0.0, https://doi.org/10.4232/1.14413, https://doi.org/10.4232/1.14454). Replication code for the analysis can be found at https://osf.io/cf35d/overview?view_only=6f6483241ecd4d2f8c8a21de4e3d2bd1.
