# Supplementary material for: Online Information as a Double-Edged Sword: A Multilevel Analysis of Vaccination Skepticism Based on ISSP 2021
Source: J Community Health. 2026 Jul 16;51(5):844–53. doi: 10.1007/s10900-026-01599-2 (PMC13614975; doi:10.1007/s10900-026-01599-2)
Supplement: Supplementary file 1 [file 10900_2026_1599_MOESM1_ESM.docx]

**Supplementary Information**

Article: Online Information as a Double-Edged Sword: A Multilevel Analysis of Vaccination Scepticism Based on ISSP 2021

Journal: Journal of Community Health

(Corresponding-)Author: Alexander Helbing, Department of Sociology, University of Technology Chemnitz, Thüringer Weg 9, 09126 Chemnitz, Germany, E-Mail: alexander.helbing@hsw.tu-chemnitz.de, Telephone: +49 371 531-39299

Author: Juliane Heise, Department of Sociology, University of Technology Chemnitz, Thüringer Weg 9, 09126 Chemnitz, Germany; Sophie Redlin, Department of Sociology, University of Technology Chemnitz, Thüringer Weg 9, 09126 Chemnitz, Germany; Peter Kriwy, Department of Sociology, University of Technology Chemnitz, Thüringer Weg 9, 09126 Chemnitz, Germany

S1: Sample by country of residence

| **Country** | **n** | **%** |
| --- | --- | --- |
| Australia | 1,050 | 2.16 |
| Austria | 1,546 | 3.19 |
| China | 2,689 | 5.54 |
| Croatia | 1,101 | 2.27 |
| Czech Republic | 1,262 | 2.60 |
| Denmark | 1,672 | 3.44 |
| Finland | 1,002 | 2.06 |
| France | 1,584 | 3.26 |
| Germany | 1,744 | 3.59 |
| Hungary | 1,008 | 2.08 |
| Iceland | 1,086 | 2.24 |
| India | 1,683 | 3.47 |
| Israel | 1,187 | 2.45 |
| Italy | 1,138 | 2.34 |
| Japan | 1,453 | 2.99 |
| Mexico | 1,001 | 2.06 |
| Netherlands | 1,269 | 2.61 |
| New Zealand | 1,135 | 2.34 |
| Norway | 1,518 | 3.13 |
| Philippines | 1,800 | 3.71 |
| Poland | 1,098 | 2.26 |
| Russia | 1,597 | 3.29 |
| Slovakia | 1,013 | 2.09 |
| Slovenia | 1,020 | 2.10 |
| South Africa | 2,829 | 5.83 |
| Spain | 2,059 | 4.24 |
| Suriname | 1,468 | 3.02 |
| Sweden | 1,930 | 3.98 |
| Switzerland | 3,349 | 6.90 |
| Taiwan | 1,604 | 3.30 |
| Thailand | 1,497 | 3.08 |
| United States | 1,146 | 2.36 |
| Sum | 48,538 | ≈ 100.0 |

Percentage does not sum to exactly 100 due to rounding errors

S2.1: Descriptive sample analysis – categorical variables

| **Variable** | **n** | **%** |
| --- | --- | --- |
| *Attitude: Vaccination do more harm than good*  Agree  Disagree & Neutral attitude | 7,173  41,076 | 14.87  85.13 |
| *Attitude: Natural immunization > vaccination*  Agree  Disagree & cannot say | 9,614  38,633 | 19.93  80.07 |
| *Internet access*  Yes  No | 44,223  4,315 | 91.11  8.89 |
| *Frequency internet usage*  Never or almost never  Several times a year  Several times a month  Several times a week  At least once a day  No internet access | 12,168  10,759  12,516  5,942  2,098  4,249 | 25.49  22.54  26.22  12.45  4.40  8.90 |
| *Digital health literacy*  Low  Neutral  High  No internet access | 28,185  11,831  6,140  2,036 | 58.48  24.55  12.74  4.22 |
| *Satisfaction healthcare system*  Not satisfied  Neutral  Satisfied | 7,099  7,782  32,705 | 14.92  16.35  68.73 |
| *Gender* Male  Female | 22,433  26,021 | 46.30  53.70 |
| *Education*  Primary education or less  Secondary education  Tertiary education | 4,952  24,969  17,832 | 10.37  52.29  37.34 |
| *Subjective health status*  Bad  Fair  Good  Very good  Excellent | 2,044  10,628  18,697  12,685  3,946 | 4.26  22.14  38.95  26.43  8.22 |
| *Survey Year*  2021  2022  2023  2024 | 26,470  12,719  4,608  4,741 | 54.53  26.20  9.49  9.77 |

Percentages do not sum to exactly 100 due to rounding errors

S2.2: Descriptive sample analysis – numerical variables

| **Variable** | **Min.** | **1^st^ Quartil** | **Median** | **3^rd^ Quartil** | **Max.** | **Mean** | **Standard Deviation** |
| --- | --- | --- | --- | --- | --- | --- | --- |
| *Age* | 16 | 35 | 50 | 64 | 111 | 49.76 | 17.49 |
| *HAQ* | 0.35 | 0.53 | 0.86 | 0.90 | 0.93 | 0.74 | 0.20 |
| *Gini* | 0.38 | 0.41 | 0.48 | 0.58 | 0.75 | 0.51 | 0.11 |

S3: Variable coding

| **Variable** | **Variable name** | **Question text and original labelling** | **Own label (original label)** |
| --- | --- | --- | --- |
| Attitude: Vaccination do more harm than good  Agree | v31 | How much do you agree or disagree with the following statements about vaccinations?  It is better to develop immunity by getting ill than having a vaccination.  -8 Can't choose  1 Strongly agree  2 Agree  3 Neither agree nor disagree  4 Disagree  5 Strongly disagree | 0=Agree (1,2)  1=Disagree (3,4,5,-8) |
| Attitude: Natural immunization > vaccination | v32 | How much do you agree or disagree with the following statements about vaccinations?  Overall, vaccinations do more harm than good.  -8 Can't choose  1 Strongly agree  2 Agree  3 Neither agree nor disagree  4 Disagree  5 Strongly disagree | 0=Agree (1,2)  1=Disagree (3,4,5,-8) |
| Internet access | v22 | During the past 12 months, how o en, if at all, did you use the internet on any device (such as computers, tablets and smartphones) to look for health or medical information for yourself or someone else?  -4=I do not have access to the internet  1=Several times a day  2=Once a day  3=Several times a week  4=Several times a month  5=Several times a year  6=Never or almost never | 0=Internet access (1,2,3,4,5,6)  1=No Internet access (-4) |
| Frequency internet usage | v25 | During the past 12 months, how often, if at all, have you used the internet to look for information on the following topics?  Information on vaccinations?  -4=NAP  -8=Can’t choose  -9=No answer  1=Never  2=Seldom  3=Sometimes  4=Often  5=Very often | 0=Never (1; Poland: v22 = 6)  1=Seldom (2)  2=Sometimes (3)  3=Often (4)  4=Very often (5)  5=No internet access (-4)  According to the missing indicator method category 5 is combined with the mode, in this case category 3  Poland filters out respondents who answer "never" to the original internet access question (v22). These cases are combined with category 0 in this analysis. |
| Digital health literacy | v30 | To what extent do you agree or disagree with the following statements?  It is not easy to distinguish between reliable and unreliable health information on the internet.  -8=Can't choose  -9=No answer  1=Strongly agree  2=Agree  3=Neither agree nor disagree  4=Disagree  5=Strongly disagree | 0=Neutral (3)  1=Low (1,2)  2=High (4,5)  3=No internet access (-9 and -8, when Internet access = -4)  According to the missing indicator method category 3 is combined with the mode, in this case category 1 |
| Gender | sex | Are you...?  1=Male  2=Female | 0=Male (1)  1=Female (2) |
| Age | age | Age is either derived from birthyear or from the following question.  What was your age at your last birthday, in full years? | Open question |
| Education | edulevel | Education is based on the following question, with an additional simplification according to ISCED 2011 scale.  What is the highest level of education you have ever completed?  0=No formal education  1=Primary education  2=Lower secondary  3=Upper secondary  4=Post secondary, non-tertiary  5=Short-cycle tertiary  6=Lower level tertiary, BA  7=Upper level tertiary, MA  8=PhD, Post Tertiary Specialization | 0=No formal education & primary education (0,1)  1=Secondary education (2,3,4)  2=Tertiary education (5,6,7,8) |
| Subjective Health | v51 | In general, would you say your health is …  1=Excellent  2=Very good  3=Good  4=Fair  5=Poor | 0=Poor  1=Fair  2=Good  3=Very good  4= Excellent |
| Satisfaction healthcare system | v44 | In general, how satisfied or dissatisfied are you with the health care system in [country]?  1=Completely satisfied  2=Very satisfied  3=Fairly satisfied  4=Neither satisfied nor dissatisfied  5=Fairly dissatisfied  6=Very dissatisfied  7=Completely dissatisfied | 0=Neutral (4)  1=Satisfied (1,2,3)  2=Dissatisfied (5,6,7) |
| Survey year | dateyr | Year of the interview (four digits)  2021  2022  2023  2024 | 0=2021  1=2022  2=2023  3=2024 |
| Country | country | The country of residence is not directly asked.  36=Australia  40=Austria  156=China  158=Taiwan  191=Croatia  203=Czech Republic  208=Denmark  246=Finland  250=France  276=Germany  348=Hungary  352=Iceland  356=India  376=Israel  380=Italy  392=Japan  484=Mexico  528=Netherlands  554=New Zealand  578=Norway  608=Philippines  616=Poland  643=Russia  703=Slovakia  705=Slovenia  710=South Africa  724=Spain  752=Sweden  740=Suriname  756=Switzerland  764=Thailand  840=United States | 0=Australia  1=Austria  2=China  3=Croatia  4=Czech Republic  5=Denmark  6=Finland  7=France  8=Germany  9=Hungary  10=Iceland  11=India  12=Israel  13=Italy  14=Japan  15=Mexico  16=Netherlands  17=New Zealand  18=Norway  19=Philippines  20=Poland  21=Russia  22=Slovakia  23=Slovenia  24=South Africa  25=Spain  26=Sweden  27=Suriname  28=Switzerland  29=Taiwan  30=Thailand  31=United States |
